# The Unrecognized Threat of Secondary Bacterial Infections with COVID-19

**DOI:** 10.1128/mBio.01806-20

**Published:** 2020-08-07

**Authors:** Mylene Vaillancourt, Peter Jorth

**Affiliations:** a Department of Pathology and Laboratory Medicine, Cedars-Sinai Medical Center, Los Angeles, California, USA

**Keywords:** COVID-19, SARS-CoV-2, antibiotic resistance, coinfection, secondary bacterial infection

## Abstract

Coronavirus disease 2019 (COVID-19) is the greatest pandemic of our generation, with 16 million people affected and 650,000 deaths worldwide so far. One of the risk factors associated with COVID-19 is secondary bacterial pneumonia. In recent studies on COVID-19 patients, secondary bacterial infections were significantly associated with worse outcomes and death despite antimicrobial therapies. In the past, the intensive use of antibiotics during the severe acute respiratory syndrome coronavirus (SARS-CoV) pandemic led to increases in the prevalence of multidrug-resistant bacteria. The rising number of antibiotic-resistant bacteria and our decreasing capacity to eradicate them not only render us more vulnerable to bacterial infections but also weaken us during viral pandemics. The COVID-19 pandemic reminds us of the great health challenges we are facing, especially regarding antibiotic-resistant bacteria.

## Abstract (precis)

Coronavirus disease 2019 (COVID-19) is the greatest pandemic of our generation, with 16 million people affected and 650,000 deaths worldwide so far. One of the risk factors associated with COVID-19 is secondary bacterial pneumonia. In recent studies on COVID-19 patients, secondary bacterial infections were significantly associated with worse outcomes and death despite antimicrobial therapies. In the past, the intensive use of antibiotics during the severe acute respiratory syndrome coronavirus (SARS-CoV) pandemic led to increases in the prevalence of multidrug-resistant bacteria.

## COMMENTARY

The outbreak of severe acute respiratory syndrome coronavirus 2 (SARS-CoV-2), which causes coronavirus disease 2019 (COVID-19), is the greatest pandemic of our generation, with 16 million people infected and 650,000 deaths worldwide so far (1). One of the great mysteries in this pandemic is why some people become critically ill while others exhibit relatively mild symptoms, even when the patients share similar risk factors.

It is becoming apparent that secondary bacterial infections occur in many COVID-19 patients and can be associated with worse outcomes. In a multicenter study that included 476 COVID-19 patients, secondary bacterial infections were significantly associated with outcome severity (2). In that study, patients were divided into 3 groups (moderately ill, severely ill, and critically ill). The critically ill patients had the highest percentage of bacterial coinfection (34.5%) compared to patients in the moderately ill and severely ill groups (3.9% and 8.3%, respectively) (2). More concerning, this higher rate of coinfections in critical patients happened although the majority of them (92.9%) received antibiotic treatments compared to 59.4% and 83.3% in the moderately ill and severely ill groups. Zhou and colleagues (3) also found that among 191 COVID-19 patients, bacterial coinfections occurred in 15% of all cases, including 50% of nonsurvivors, even though 95% of patients received antibiotics. Even more troubling, 27/28 COVID-19 patients with coinfections succumbed (3). In both studies, other comorbidities were also associated with mortality; thus, it is difficult to determine the exact impact of coinfections. A third study (4) used real-time PCR to detect specific pathogens causing COVID-19 coinfections. They found that 243 (94.2%) patients were coinfected with at least 1 of 39 different pathogens. Bacterial coinfections were predominant (91.8%) over viral (31.5%) and fungal (23.3) infections. Although the authors found no significant association between coinfection rates and outcome severity or mortality, they described interesting coinfection patterns in different clinical groups (asymptomatic and mildly, moderately, and severely/critically ill). For instance, Streptococcus pneumoniae, Klebsiella pneumoniae, Haemophilus influenzae, Escherichia coli, Staphylococcus aureus, *Aspergillus*, and Epstein-Barr (EB) virus were detected in all four clinical groups, while Pseudomonas aeruginosa, human adenovirus, human rhinovirus, and herpes simplex virus were detected only in symptomatic patients regardless of disease severity. Interestingly, coinfections with influenza A virus, influenza B virus, or coronavirus were not common in these COVID-19 patients, though samples were collected during the flu season (4). Altogether, these early data suggest that the specific coinfecting pathogens may worsen disease prognosis and warrant further investigation.

While it is unclear whether coinfections definitively worsen COVID-19 patient outcomes, historical data from pandemics and seasonal flu suggest that bacterial coinfections can worsen viral diseases (5–13). During the first SARS-CoV outbreak in 2003, up to 30% of patients were diagnosed with secondary bacterial infections and coinfection was positively associated with disease severity (5, 6). Bacterial coinfections are also present during regular influenza seasons in 2% to 65% of cases and are associated with morbidity and mortality (7–9). Moreover, during the flu seasons between 2004 and 2007 in the United States, bacterial coinfection rates in children dangerously increased from 6% (2004 to 2005) to 15% (2005 to 2006) and 34% (2006 to 2007) (9). The increasing rates of bacterial coinfections occurring during regular flu seasons highlight the urgent need to investigate this phenomenon more extensively, especially as it relates to COVID-19.

We are using more antibiotics in our fight to save COVID-19 patients from bacterial coinfections, and it is important to consider how this could affect the prevalence of antibiotic-resistant bacteria globally. During the first SARS-CoV outbreak, analyses of isolates collected from patients in the intensive care unit (ICU) in Prince of Wales Hospital (Hong Kong) from 12 March to 31 May 2003 showed that rates of methicillin-resistant S. aureus acquisition drastically increased during the outbreak from 3.53% pre-SARS to 25.30% during the SARS outbreak, despite extensive infection control precautions (10). Other pathogens were found in postmortem lung specimens of patients from Hong Kong and Singapore, including S. aureus, P. aeruginosa, *Klebsiella* spp., and S. pneumoniae, all of which are well known for their high resistance to a broad spectrum of drugs (14, 15). It is not clear whether the COVID-19 outbreak will lead to increased rates of antibiotic-resistant bacteria since the use of antibiotics does not always result in increased rates of drug-resistant strains (16), yet it will be important to continue monitoring rates of antibiotic-resistant bacterial infections.

These data from the current COVID-19 pandemic, previous pandemics, and seasonal influenza raise important questions that need to be investigated. First, are there synergic interactions between the SARS-CoV-2 virus and certain coinfecting bacteria? Second, does coinfection with antibiotic-resistant bacteria affect disease severity? Indeed, some of the pathogens detected in COVID-19 patients can be antibiotic resistant, which could reduce the efficacy of treatments administered to patients. Unfortunately, in the first two studies, where coinfections were associated with worse outcomes (2, 3), the specific coinfecting pathogens detected were not described and no studies thus far have analyzed rates of coinfection by antibiotic-resistant bacteria. Thus, it is impossible to determine from the available data whether certain bacterial species or whether antibiotic-resistant strains correlate with outcome severity or mortality. However, the presence of antibiotic-resistant bacteria could potentially explain the high rates of bacterial coinfections in critically ill patients despite extensive antibiotic treatments in these cohorts. Finally, the battle with COVID-19 may accelerate the worsening of our already dire situation with respect to antibiotic-resistant pathogens. The rising number of multidrug-resistant bacteria and our decreasing capacity to eradicate them not only render us more vulnerable to bacterial infections but also weaken us during viral pandemics. To tackle this serious issue, we urgently need to investigate the effects of bacterial coinfections during viral infections and find new antimicrobial compounds to eradicate multidrug-resistant pathogens.
